# A computational model of the temporal dynamics of plasticity in procedural learning: sensitivity to feedback timing

**DOI:** 10.3389/fpsyg.2014.00643

**Published:** 2014-07-02

**Authors:** Vivian V. Valentin, W. Todd Maddox, F. Gregory Ashby

**Affiliations:** ^1^Department of Psychological and Brain Sciences, University of California Santa BarbaraSanta Barbara, CA, USA; ^2^Department of Psychology, University of Texas AustinAustin, TX, USA

**Keywords:** feedback timing, procedural learning, striatum, computational modeling, category learning, synaptic plasticity, dopamine

## Abstract

The evidence is now good that different memory systems mediate the learning of different types of category structures. In particular, declarative memory dominates rule-based (RB) category learning and procedural memory dominates information-integration (II) category learning. For example, several studies have reported that feedback timing is critical for II category learning, but not for RB category learning—results that have broad support within the memory systems literature. Specifically, II category learning has been shown to be best with feedback delays of 500 ms compared to delays of 0 and 1000 ms, and highly impaired with delays of 2.5 s or longer. In contrast, RB learning is unaffected by any feedback delay up to 10 s. We propose a neurobiologically detailed theory of procedural learning that is sensitive to different feedback delays. The theory assumes that procedural learning is mediated by plasticity at cortical-striatal synapses that are modified by dopamine-mediated reinforcement learning. The model captures the time-course of the biochemical events in the striatum that cause synaptic plasticity, and thereby accounts for the empirical effects of various feedback delays on II category learning.

## Introduction

Learning, by definition, is a process of laying down a new memory trace, or of strengthening an existing trace. For this reason, learning and memory are inextricably related. It is now widely accepted that humans have multiple memory systems (Cohen et al., 1985; Squire et al., 1993; Schacter and Wagner, 1999), and not surprisingly, evidence is also building that humans have multiple learning systems (Sloman, 1996; Ashby et al., 1998; Erickson and Kruschke, 1998). The different learning and memory systems that have been identified are mostly mediated by separate neural systems and have qualitatively different properties.

One major difference among learning and memory systems concerns the role of feedback. For example, procedural learning appears impossible without trial-by-trial feedback (e.g., Ashby et al., 1999), whereas the perceptual representation memory system does not depend on feedback for learning. Instead, simple repetition is sufficient (e.g., Schacter, 1994; Wiggs and Martin, 1998). In contrast, in declarative memory systems, feedback plays a facilitative role in the sense that it often improves learning, but is sometimes not necessary at all (e.g., Ashby et al., 1999). Procedural and declarative memory systems also differ with respect to their sensitivity to the timing of feedback. Learning in tasks that depend on declarative memory is flexible with regards to feedback timing, in the sense that long timing delays often have no detrimental effect on learning. In contrast, for procedural learning, the timing of feedback is critical. Learning is best when feedback immediately follows the behavior. The importance of immediate feedback has been documented in many operant conditioning studies in the animal literature. One of the earliest and most influential of these showed a deficit in conditioning to lever-press with delayed reinforcement (Skinner, 1938; pp. 72–74).

Perhaps the best known example of procedural learning is the learning of motor skills (Willingham, 1998). Even so, the evidence is good that some cognitive skills are acquired procedurally, including certain types of categorization (Ashby et al., 1998; Maddox and Ashby, 2004; Ashby and Maddox, 2005, 2010). One categorization task that is known to depend on procedural learning is the information-integration (II) task. In II tasks, stimuli are assigned to categories in such a way that accuracy is maximized only if information from two or more non-commensurable stimulus dimensions is integrated at some predecisional stage (Ashby and Gott, 1988). Typically, the optimal strategy in II tasks is difficult or impossible to describe verbally. II tasks are often contrasted with rule-based (RB) categorization tasks. In RB tasks, the categories can be learned via some explicit reasoning process. Frequently, the rule that maximizes accuracy is easy to describe verbally (e.g., as when only a single separable dimension is relevant). A large literature implicates declarative memory systems, and especially working memory and executive attention, in RB tasks (Waldron and Ashby, 2001; Maddox et al., 2004; Zeithamova and Maddox, 2006, 2007).

**Figure 1:**
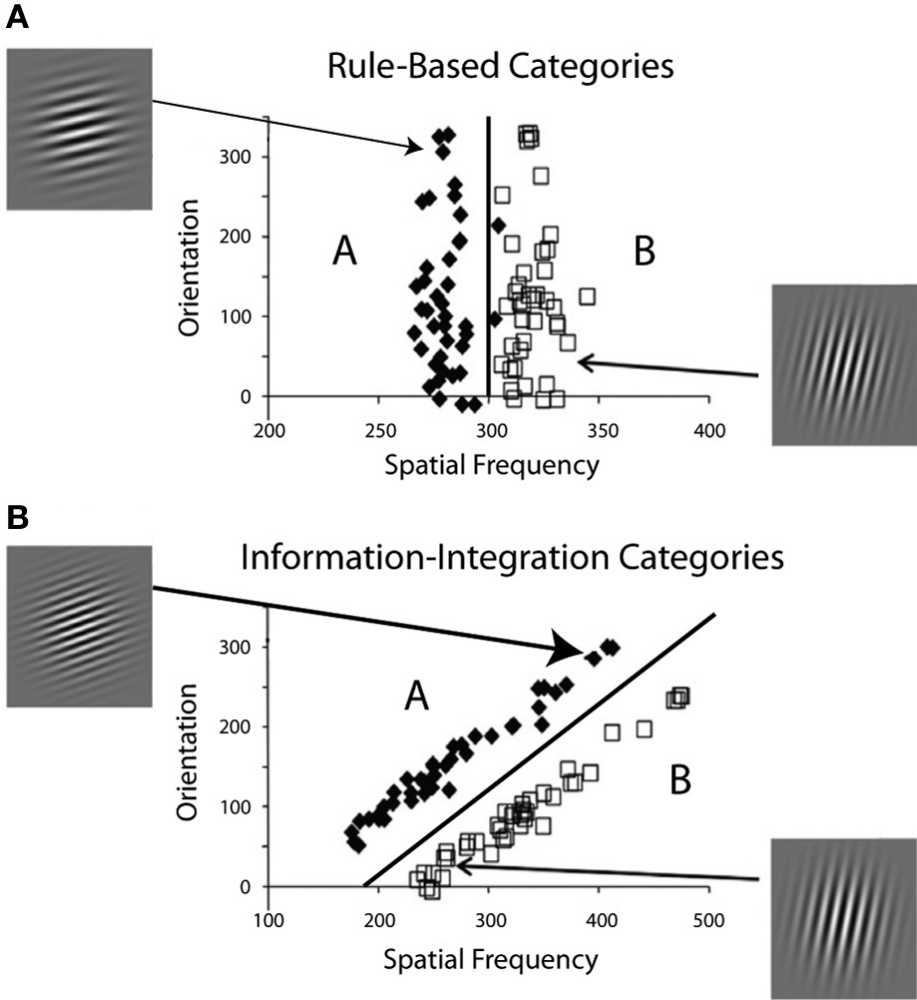
**Two category structures used in the experiments, along with sample stimuli from each category. (A)** A one-dimensional rule-based structure, and **(B)** a two-dimensional information-integration structure.

Figure 1 shows examples of RB and II category-learning tasks. In both tasks each stimulus in the two contrasting categories is a sine-wave grating. All stimuli have the same size, shape, and contrast and differ only in bar width and bar orientation. In a typical application, a single stimulus is shown on each trial and the participant's task is to assign the stimulus to its correct category. Feedback about the accuracy of the response is then given after some delay.

Several studies have shown that RB learning is unaffected by feedback delays as long as 10 s (Maddox et al., 2003; Maddox and Ing, 2005), which fits well with the theory that declarative and working memory systems are recruited. For example, when an explicit rule is used to make a categorization response, it can be maintained in working memory during feedback delays. In contrast, II categorization, which is thought to depend on procedural learning, is highly impaired with delays of 2.5 s or longer (Maddox et al., 2003; Maddox and Ing, 2005; Dunn et al., 2012). In these experiments, a mask that was visually similar to the stimulus was presented during the delay period (i.e., during the time between response and feedback) in order to prevent visual imagery from maintaining a trace of the stimulus during the delay. When a mask is used that is visually dissimilar to the stimulus, II learning remains compromised by feedback delays, but by a reduced amount (10% instead of the 20% accuracy deficit with a similar mask; Dunn et al., 2012). This may be because the visual imagery during the delay period lends itself to an additional declarative memorization strategy. A full feedback procedure (when the correct category is indicated after an incorrect response) has a similar effect of reducing the feedback delay deficit on II learning (Dunn et al., 2012, Experiments 3 and 4). This may also be because additional declarative mechanisms may be recruited during learning, because full feedback has been shown to facilitate the use of verbal rules in both RB and II tasks (Maddox et al., 2008). However it is beyond the scope of this paper to discuss experimental manipulations in which multiple learning mechanisms might be operating. Therefore we will focus on experiments in which feedback is minimal (simply “correct” or “incorrect”), as opposed to a full feedback procedure. In an experiment without masks and with minimal feedback, II learning was best with feedback delays of 500 ms and slightly worse with delays of 0 or 1000 ms (Worthy et al., 2013). This complex pattern of results suggests that there is an optimal time frame for feedback to arrive after a response. This article describes a biologically detailed computational model of procedural learning that accounts for the effects of these various feedback delays.

Much evidence suggests that procedural learning is mediated largely within the striatum, and is facilitated by a dopamine (DA) mediated reinforcement learning signal (Knopman and Nissen, 1991; Grafton et al., 1995; Jackson and Houghton, 1995; Badgaiyan et al., 2007). The well-accepted theory is that positive feedback that follows successful behaviors increases phasic DA levels in the striatum, which has the effect of strengthening recently active synapses, whereas negative feedback causes DA levels to fall below baseline, which has the effect of weakening recently active synapses. In this way, the DA response to feedback serves as a teaching signal for which successful behaviors increase in probability and unsuccessful behaviors decrease in probability. According to this account, synaptic plasticity (long term potentiation, LTP, or long term depression, LTD) can only occur when the visual trace of the stimulus and the post-synaptic effects of DA overlap in time.

The cortical excitation induced by the visual stimulus results in glutamate release into the striatum, which initiates several post-synaptic intracellular cascades that alter the cortical-striatal synapse (e.g., Rudy, 2014). One such cascade, which seems especially important for cortical-striatal synaptic plasticity, is mediated by NMDA receptor activation and results in the phosphorylation of calcium/calmodulin-dependent protein kinase II (CaMKII; e.g., Lisman et al., 2002). During a brief period of time (thought to be several seconds), when CaMKII is partially phosphorylated, a chemical cascade^1^ that is initiated when DA binds to D1 receptors can potentiate the LTP-inducing effects of CaMKII (e.g., Lisman et al., 2002). Thus, the effects of feedback should be greatest when the peak effects of the DA-induced cascade overlap in time with the period when CaMKII is partially phosphorylated. We know of no data as to the exact time-course of these events, but it must take some time (on the order of milliseconds) for both cascades to escalate to a peak and then gradually to decline. The further apart in time these two cascades peak, the less effect DA will have on synaptic plasticity. This model provides a biological constraint on the time of optimal feedback delivery. In summary, the theory proposed here assumes that optimal procedural learning occurs when stimulus and feedback driven events within the striatum peak simultaneously, which can only happen if feedback is given several hundred milliseconds (i.e., 500 ms) after a response to the stimulus has been made.

## Theoretical analysis

This section describes a computational cognitive neuroscience model of procedural category learning that we developed to formally test hypotheses about various feedback delays^2^. There are two components to the model, (1) a procedural category-learning network, and (2) a reward-learning algorithm that predicts DA release.

### Procedural category-learning model

**Figure 2:**
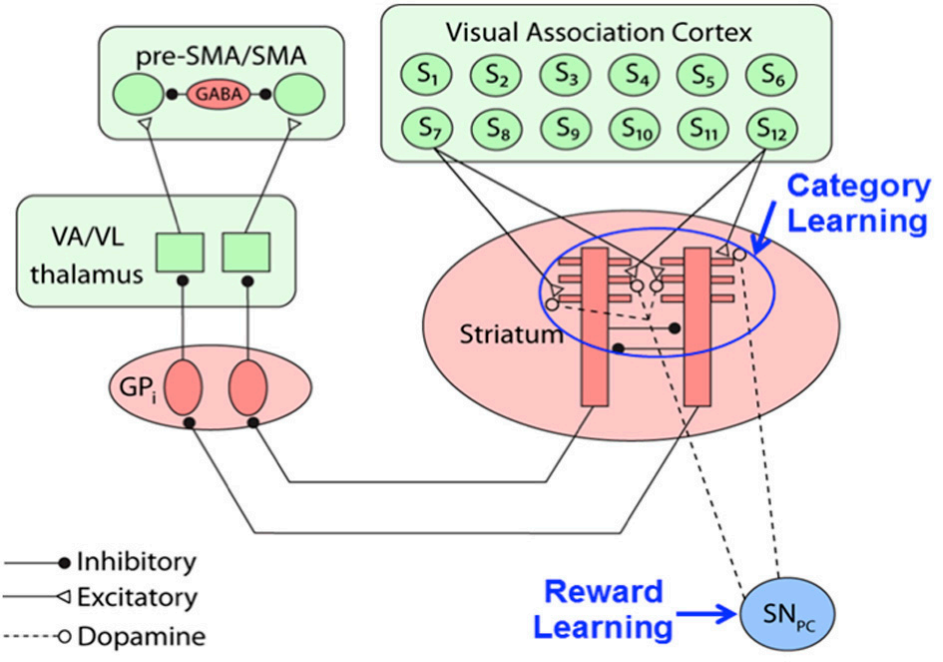
**Procedural category learning network**. Dopamine release from the substantia nigra pars compacta (*SN_PC_*) leads to synaptic strengthening of cortical-striatal synapses activated by presentation of a visual stimulus, *S_i_*. (GP_i_, internal segment of the globus pallidus; VA/VL, ventral anterior/ ventral lateral nuclei of the thalamus; SMA, supplementary motor area).

The basic architecture of the category learning portion of our model, shown in Figure 2, is a simplified version of a model proposed by Ashby and Crossley (2011). For more details, see the Supplementary Material, but briefly, the model is a distributed network of spiking neurons generated from differential equations. The model of the striatal medium spiny neurons (MSNs) was adapted from a model proposed by Izhikevich (2007; p. 312). The key inputs to the MSNs include excitatory inputs from sensory cortex and inhibitory input from other MSNs. For all other units in the network, we model the membrane potential with the standard quadratic integrate-and-fire model (Ermentrout, 1996). The globus pallidus internal segment (GPi) units receive inhibitory inputs from the MSNs, which release the thalamus from GPi's tonic inhibition, freeing the thalamus to send excitatory inputs to the premotor units, which laterally inhibit each other. The premotor unit that passes an activation threshold first selects the category response. If neither unit crosses the threshold or if the units are equally active, the response is randomly selected.

The Figure 2 model assumes that category learning is mediated via synaptic plasticity at cortical-striatal synapses. Following standard models, we assume that synaptic plasticity at all cortical-striatal synapses is modified according to reinforcement learning that requires three factors: (1) strong presynaptic activation, (2) postsynaptic activation that is strong enough to activate NMDA receptors, and (3) DA levels above baseline (Calabresi et al., 1996; Arbuthnott et al., 2000; Reynolds and Wickens, 2002). If any of these conditions are absent then the synapse is weakened. More specifically, let *w_K,J_*(*n*) denote the strength of the synapse on trial *n* between cortical unit *K* and striatal unit *J*. Following Ashby and Crossley (2011), our reinforcement learning model assumes:

(1) $$\begin{array}{l} w_{K,J}(n+1)=w_{K,J}(n)+\;\alpha_{w}I_{K}(n){\left[S_{J}D(n)-D_{\text{base}}\right]}^{+}\left[1-w_{K,J}(n)\right]\\ \;-\;\beta_{w}I_{K}(n){\left[D_{\text{base}}-S_{J}D(n)\right]}^{+}w_{K,J}(n)\\ \;-\;\gamma_{w}I_{K}(n){\left[\theta_{\text{NMDA}}-S_{J}(n)\right]}^{+}{\left[S_{J}(n)-\theta_{\text{AMPA}}\right]}^{+}w_{K,J}(n) \end{array}$$

The function [*g*(*n*)]^+^ = *g*(*n*) if *g*(*n*) > 0, and otherwise *g*(*n*) = 0. *I_K_* is the input activation from cortical unit *K*, the constant *D*_base_ is the baseline DA level, the product, *S_J_D*(*n*), is the magnitude of DA's postsynaptic potentiating effect [*D*(*n*)] on the unit *J* MSN (*S_J_*) on trial *n*, and α_*w*_, β_*w*_, γ_*w*_, θ_NMDA_, and θ_AMPA_ are all constants. The first three of these (i.e., α_*w*_, β_*w*_, and γ_*w*_) operate like standard learning rates because they determine the magnitudes of increases and decreases in synaptic strength. The constants θ_NMDA_ and θ_AMPA_ represent the activation thresholds for postsynaptic NMDA and AMPA (more precisely, non-NMDA) glutamate receptors, respectively. The numerical value of θ_NMDA_ > θ_AMPA_ because NMDA receptors have a higher threshold for activation than AMPA receptors. This is critical because NMDA receptor activation is required to strengthen cortical-striatal synapses (Calabresi et al., 1992).

The first line in Equation 1 describes the conditions under which synapses are strengthened (i.e., striatal activation above the threshold for NMDA receptor activation and DA above baseline) and lines two and three describe conditions that cause the synapse to be weakened. The first possibility (line 2) is that postsynaptic activation is above the NMDA threshold but DA is below baseline (as on an error trial), and the second possibility is that striatal activation is between the AMPA and NMDA thresholds. Note that synaptic strength does not change if postsynaptic activation is below the AMPA threshold.

### Reward learning model

Note that Equation 1 requires a model that specifies exactly how much DA is released on each trial. We model DA neuron firing as we did the MSNs, except with constants selected by Izhikevich (2007) to mimic a regular spiking neuron. The other difference is in the input. For DA neurons, the input comes from a complex and widely distributed network that likely includes areas in frontal cortex, the amygdala, the ventral striatum, and the pedunculopontine tegmental nucleus. Models of this network exist (e.g., Brown et al., 1999), but we make no attempt to model this network in a biologically detailed way. Our goal is to understand how procedural learning occurs under a variety of feedback delays. So we provide a detailed model of the procedural learning network, but not of the reward-learning network that provides the feedback-based input to the procedural-learning network. However, we will model the processing that occurs in the reward-learning network at a more abstract level.

Much evidence suggests that the DA response to the feedback increases with the reward prediction error (*RPE*), and the DA response to cues that predict reward increase with the probability of future reward (reward prediction, *RP*; Schultz, 1998, 2002, 2006; Schultz et al., 1998). *RPE* is defined as the value of obtained reward (*R*, 1 if correct, and 0 if incorrect) minus the value of the predicted reward (i.e., the RP) on trial *n*:

$$RPE_{n}=R_{n}-RP_{n}$$

We used the single-operator learning model (Bush and Mosteller, 1951) to update *RP_n_* for each trial:

$$RP_{n}=RP_{n}+\alpha_{pr}(RPE_{n})$$

The learning rate, α_*pr*_ was set to 0.075. This model predicts that *RP_n_* will converge exponentially to the true expected reward value and then fluctuate around this value until reward contingencies change.

*RP_n_* and *RPE_n_* serve as the inputs in the DA spiking equations. The effects of DA on synaptic plasticity however, are not due directly to the firing of DA neurons, but instead to the interaction between post-synaptic effects produced by firing in the cortical glutamate and DA neurons. Following glutamate release due to cortical excitation, various slow postsynaptic biochemical events are initiated in the MSN. One important example occurs when glutamate activates postsynaptic NMDA receptors. As mentioned earlier, this initiates several chemical cascades within the MSN, which result in partial phosphorylation of CaMKII. When fully phosphorylated, CaMKII initiates structural changes that have the effect of strengthening the cortical-striatal synapse. DA can potentiate the phosphorylation of CaMKII (by a cascade of events that follow D1 receptor binding), but only if the DA levels increase at the appropriate time—that is, when the CaMKII is partially phosphorylated (Hemmings et al., 1984; Halpain et al., 1990). We model the postsynaptic effects of DA and glutamate via the alpha function (see Supplementary Material). More specifically, the postsynaptic effects of excitation in either the DA neurons or the sensory cortical neurons are delayed and smeared out in time via this function. The time-course of these two alpha functions is critical because DA can only potentiate the post-synaptic effects of glutamate. Thus, synaptic modification can only occur when the DA alpha function (i.e., the DA trace) and the glutamate alpha function (i.e., the glutamate trace) overlap. The amount of overlap is specified by the term *S_J_D*(*n*) in Equation 1.

**Figure 3:**
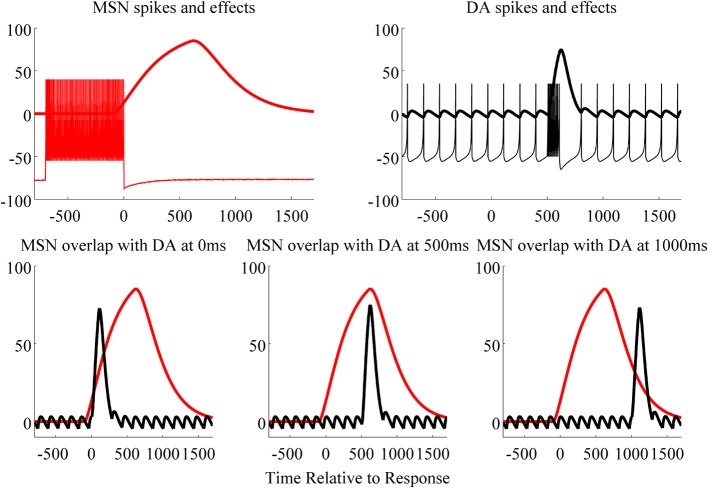
**Top:** MSN spikes and postsynaptic effects of stimulus presentation (response terminated after 700 ms; left), and DA spikes and postsynaptic effects for a correct feedback (delayed 500 ms post response/stimulus, right). The x-axis indicates the time from response (set to 0): negative values are before response, and positive values are after response in milliseconds. **Bottom**: the overlap of the MSN and DA effects is greatest when feedback is delayed 500 ms (middle) compared to 0 (left) and 1000 ms (right). The larger the overlap, the larger the product of these two curves. The integral under the product curve is proportional to the change in synaptic weight for the stimulus given a specific delay.

The top left panel of Figure 3 shows spikes (action potentials) from the glutamate activated MSN and the postsynaptic effects of glutamate (which we hereafter refer to as the glutamate trace; i.e., modeled via alpha functions). The top right panel of Figure 3 shows spikes from a substantia nigra DA neuron and the postsynaptic effects of DA. Our model of the glutamate-trace included a 550 ms lag (with decay, λ, set to 200). This lag was a free-parameter in the model, and was fixed to this value because it yielded optimal accuracy for conditions in which feedback was delayed by 500 ms. In contrast, the DA-trace included no lag and a quicker decay (i.e., λ = 100). The glutamate-trace may be delayed because of the time it takes for MSN dendrites to be depolarized, which must occur before induction of synaptic plasticity is possible. Evidence suggests that the glutamate trace may be slower to rise than the DA trace, either because of the contribution of backpropagating action potentials and/or the generation of a plateau potential (up-states) due to convergent synaptic inputs (Surmeier et al., 2009). Note that the overlap between the glutamate and the DA traces (alpha functions) is greatest when feedback-induced DA release starts at 500 ms after the response terminated stimulus display (bottom panels of Figure 3; time of response is marked by 0). The greater the overlap, the greater the product of the glutamate and DA traces. We assume that *S_J_D*(*n*) equals the area under the product curve, and therefore that this area determines how much the synaptic weight is changed on each trial.

## Methods and results of the behavioral experiments modeled

In each condition of all experiments, participants learned two categories composed of Gabor patches (sine wave gradients modulated by a circular Gaussian function) that varied across trials in spatial frequency and spatial orientation. The RB and II category structures and examples of some stimuli are shown in Figure 1. The basic trial design was the following: the stimulus was displayed on each trial until the participant responded with a category label (i.e., “A” or “B”), which was followed by corrective feedback. The delay between response and feedback varied across conditions and experiments. We define the feedback delay as the time between the response/stimulus offset and the feedback display. To analyze the data, the accuracy, and the best-fitting decision strategy in each of the 80-trial blocks were determined for each participant of all experiments.

Decision-bound modeling was used for the strategy analysis (Maddox and Ashby, 1993). The results indicate whether each participant's responses are more consistent with an explicit, rule-based strategy, with a procedural strategy, or with random guessing. As expected, more participants appeared to use procedural strategies in the shorter delay conditions than when the feedback delay was long. Even so, many participants who failed to adopt a procedural strategy showed strong evidence of rule use rather than guessing, perhaps because even simple one-dimensional rules lead to higher accuracy than guessing.

### Maddox et al. (2003)

**Figure 4:**
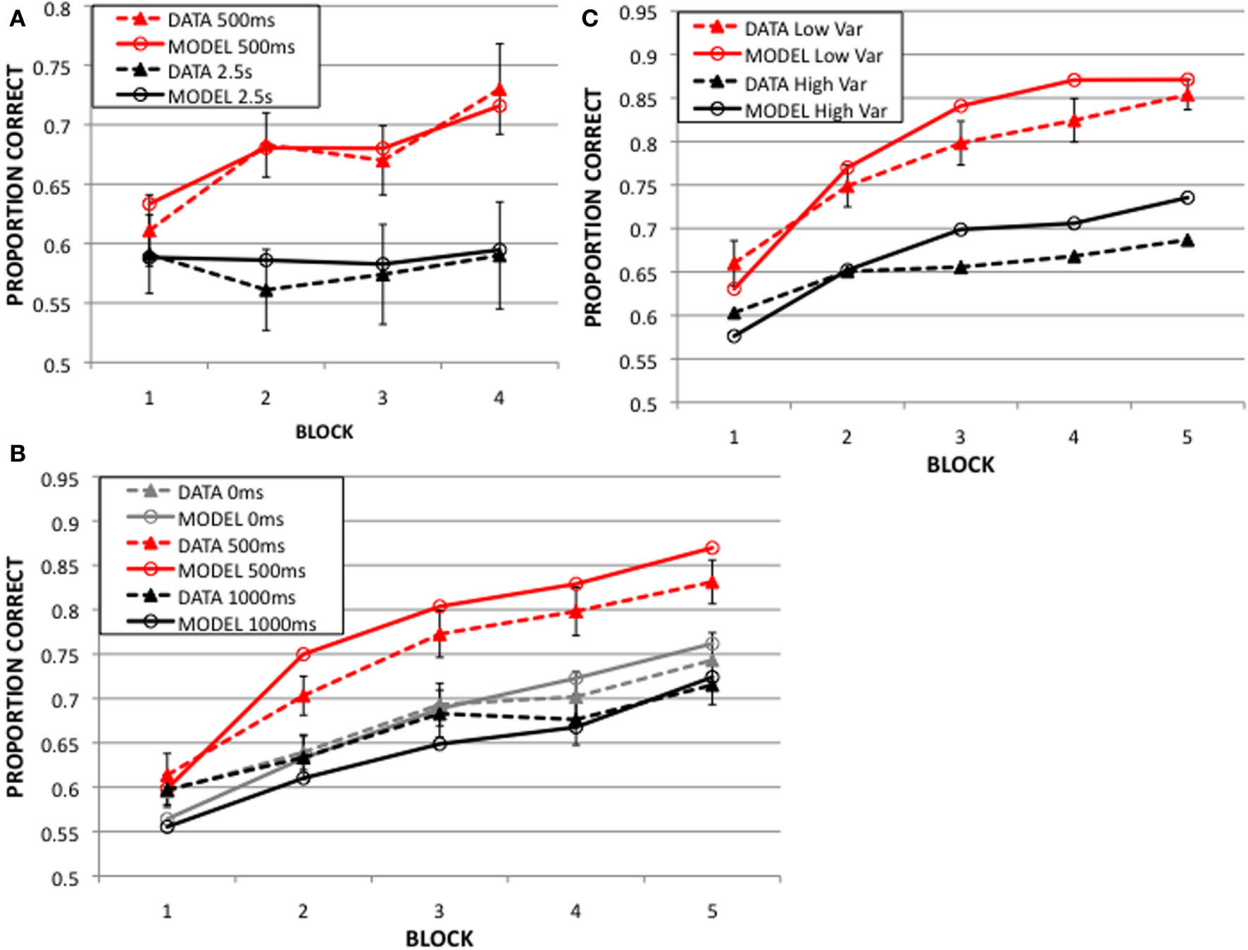
**Dynamic procedural category learning model results from 50 simulations of:** **(A)** 2003 Experiment: feedback delay of 500 ms yields much better learning than that of 2.5 s. **(B)** 2013 Experiment 1: learning is best at 500 ms feedback delay, and worse at 0 s and 1 s delays. **(C)** 2013 Experiment 2: with a mean of 500 ms feedback delay, low variance around the mean yields better learning than high variance.

In the “immediate feedback” conditions, the feedback delay was 500 ms, and in the “delayed feedback” conditions the feedback delay was much longer; 2.5, 5, or 10 s. A visual mask was presented during the feedback delay in order to minimize visual imagery. The solid lines in Figure 4A display the mean accuracies in the immediate and delayed conditions in each of the 4 blocks (ignore the dashed lines for now). Note that accuracy increased with practice when the delay was 500 ms, but there was no evidence of learning with the long delay.

### Worthy et al. (2013; experiment 1)

The feedback delay was 0, 500, or 1000 ms, depending on the condition. There was no visual mask presented during the feedback delay in this experiment because there would have been no way to present a mask in the 0ms feedback condition. Figure 4B shows that accuracy increased in all conditions, but performance was best when the delay was 500 ms condition.

### Worthy et al. (2013; experiment 2)

This experiment used delays of random duration, but with a mean of 500 ms. In the low variance condition the feedback delays had a standard deviation of 75 ms, and in the high variance condition the standard deviation was 150 ms. A larger proportion of the trials are around the optimal 500 ms feedback delay in the low compared to the high variance condition. Figure 4C shows that accuracy increased for both conditions, but performance was better in the low than the high variance condition.

## General modeling methods

Visual cortex was modeled as a rectangular grid of units, each maximally sensitive to a specific spatial frequency and orientation. On each trial, one stimulus from the Figure 1 categories was sampled and presented to the model. Visual receptive fields were modeled with a Gaussian filter that was centered on the unit tuned to the stimulus. The filter had a height of 600 and a spread of 0.8. The height of the filter at each unit determined its activation level. Thus, units tuned to perceptually similar stimuli were also activated. All differential equations were solved numerically using Euler's method.

The model responded A or B depending on whether the output from premotor unit A or premotor unit B first crossed a threshold of 5. If the output from neither premotor unit crossed the threshold, or both units crossed the threshold at exactly the same time, then the model randomly selected between A and B. All activated weights were updated on each trial by the learning equations. Predicted accuracy was computed for each block based on the proportion of correct responses of the model.

As mentioned above, some participants used explicit, rule-based strategies rather than procedural strategies, and a few participants randomly guessed. Furthermore, the number of participants who did not use a procedural strategy varied across experiments and conditions. The model is constrained to always use a procedural strategy, so predicted accuracy from the model was used to account for the responses of all participants whose data were best fit by a decision bound model that assumed a procedural strategy. We assumed that the accuracy of guessers was 0.5, and that the accuracy of rule users was equal to the best possible accuracy of a one-dimensional rule in the presence of perceptual and criterial noise^3^. In other words, suppose the decision bound modeling indicated that 15 participants in some condition used a procedural strategy, 4 participants used an explicit rule, and 1 guessed randomly. Then the average accuracy across all participants predicted by our modeling approach for this condition was

        (0.75 × accuracy of procedural model)

        +(0.20 × accuracy of best 1D rule) + (0.05 × 0.50).

Note that using this method to model the accuracy of participants who failed to use a procedural strategy adds no free parameters to our overall model. Another possible approach is to delete the data of participants who did not use a procedural strategy, and therefore exclude them from the modeling^4^. The weakness of this approach is that the number of participants remaining may be very small in some conditions and early in learning, and individuals may switch strategies from block to block even late in learning. The number of participants who adopt a procedural strategy increases with training; therefore it is most meaningful to model their accuracy in the last block. The data from the three experimental results were each modeled with the mean of 50 independent replications.

The numerical values of all parameters in the category-learning model were set to the values used by Izhikevich (2007; p. 312) and Ashby and Crossley (2011). Thus, the only parameters that were manipulated for the simulations described in this article were parameters from the learning equations (Equation 1), the lag for generating the MSN alpha function, and the noise variance in the premotor units (σ_C_, to account for noise from a mask in the 2003 experiment). Except for σ_C_, all free parameters were held constant across all three experiments. The parameters were estimated via a course grid search of the parameter space (values are given in Table A1). No attempt was made to optimize goodness-of-fit. However, it is important to note that similar models are highly insensitive to small or moderate changes in parameter values (Ashby and Crossley, 2011; Helie et al., 2012a,b). This makes it highly likely that results from an optimized search would not differ significantly from the results presented here.

## Modeling results

Figure 4 shows the predictions of the model (dashed lines) along with the corresponding behavioral data (solid lines). Note that the model nicely captures the qualitative properties of the data. First, both the model and humans learn best when the feedback delay is 500 ms. Second, neither shows any evidence of learning at the longest delay (i.e., 2.5, Figure 4A). Third, the model correctly predicts that immediate feedback (0 ms) comes too soon and feedback at a 1s delay is too late for optimal learning (Figure 4B). Finally, when the feedback delay is random, the model and humans both learn better with the smaller variance than with the larger variance (Figure 4C). The quantitative fit is also impressive. In fact, the model successfully accounts for 96.5, 97.2, and 93.5% of the variance in the data in Figures 4A–C, respectively.

Note that accuracy in the 500 ms delay condition (2003 Experiment) is lower (73%) than in the 2013 experiments (80%). This is likely because the 2003 Experiment included a mask (a visually similar stimulus) during the delay between response and feedback to minimize visual imagery of the categorized stimulus. Adding the mask most likely resulted in an additional source of noise, which we modeled by increasing the noise variance in the premotor (response) unit (σ_C_ in Equation A.6, see Table A1 in the Supplementary Material). By allowing this additional free parameter, the prediction matched the 500 ms delay data in the 2003 Experiment (Figure 4A).

**Figure 5:**
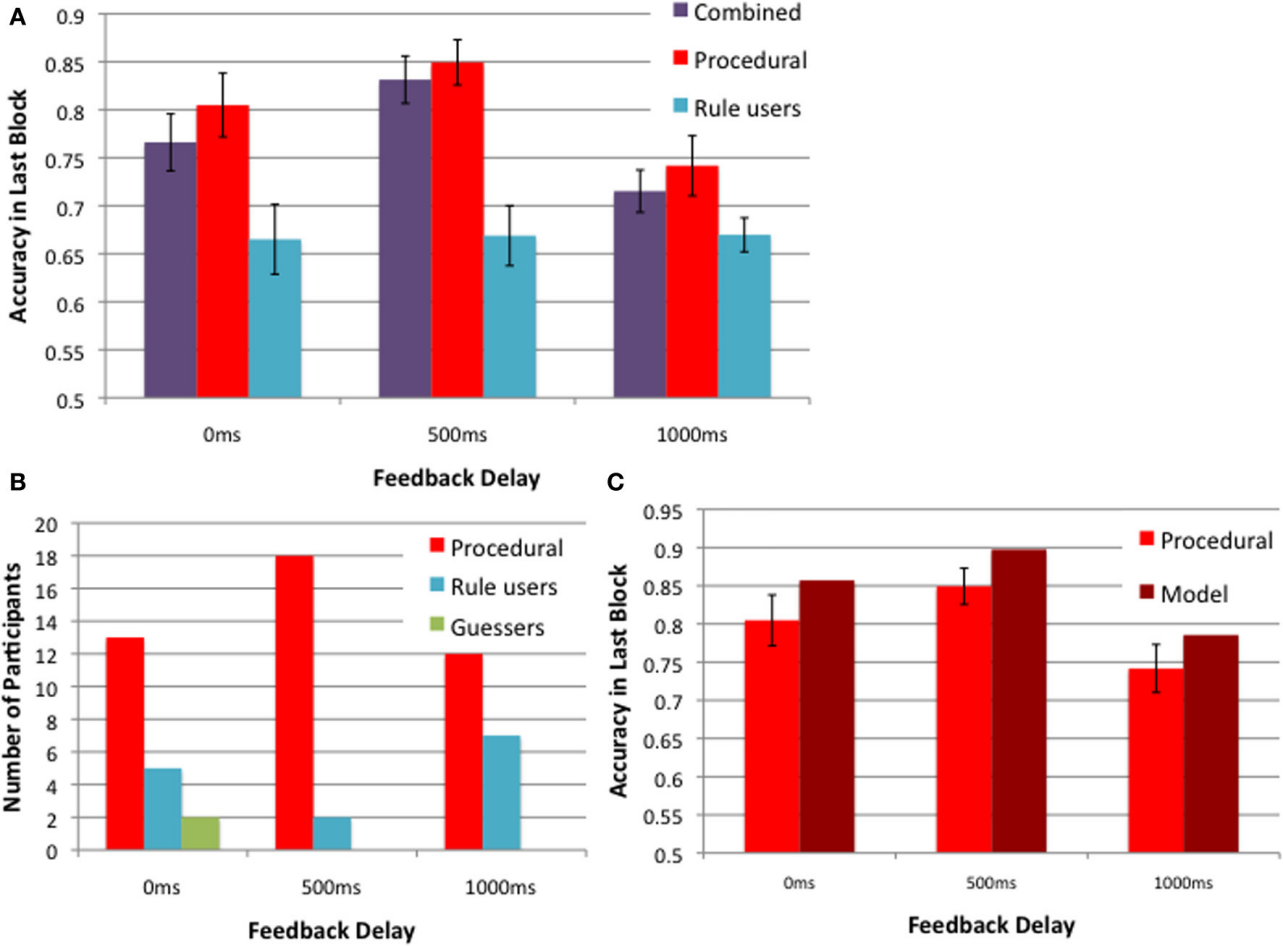
**2013 Experiment 1 strategies:** **(A)** feedback delay of 500 ms yields better learning than that of 0 and 1000 ms for a group of participants using a procedural strategy (in red), but not for those using rules (in blue). The combined accuracies for these two groups are in purple. **(B)** The numbers of participants in these two groups and an additional group of guessers (in green). **(C)** The model predictions from 50 simulations and the corresponding data of participants using the procedural strategy in the 5th block of the 0, 500, and 1000 ms feedback delay conditions.

Figure 5 further explores the 2013 Experiment 1 data, based on strategies. Figure 5A shows the block 5 accuracies in the 0, 500, and 1000 ms delay conditions for all participants (in purple), and these same data broken down into two groups based on whether the best-fitting decision bound model assumed explicit rule use or a procedural strategy. Note that the accuracy of procedural participants (in red) is greatest when the delay is 500 ms, but the accuracy of rule users (blue) is unaffected by feedback delay. Therefore whether the task is II or RB, and whether rule use is suboptimal or optimal, respectively, learning appears to be unaffected by the length of the feedback delay when participants use rules. Figure 5B shows the number of participants whose responses were best fit by a procedural strategy (in red), explicit rule (in blue), and guessing (in green). More participants used a procedural strategy when the feedback delay was 500 ms compared to 0 or 1000 ms. Even so, note that the number of rule users is small for any strong conclusions to be drawn about their insensitivity to feedback delays. On the other hand, the number of participants using a procedural strategy may be sufficient for modeling. Figure 5C shows the model predictions alongside the data of participants who used a procedural strategy in the 5th block across the 3 delay conditions. As when the data from all participants were modeled together (Figure 4B), the model nicely accounts for the empirical effects of feedback delay (Figure 5C). The only misprediction is that the model is slightly more accurate than the participants for all delays. But recall that the model used a procedural strategy on every trial of the experiment, whereas the participants presumably began with explicit strategies and only switched to a procedural strategy sometime before the last block.

## General discussion

We developed a neurobiologically-detailed computational model that successfully accounts for the effects of varying the feedback delay on procedural category learning. In line with the results from three behavioral experiments (Maddox et al., 2003; Worthy et al., 2013), we show that the model predicts that learning is best with a 500 ms feedback delay, somewhat worse for 0 and 1 s delays, and completely absent with long (i.e., 2.5 s) delays. The successful fits suggest that the temporal dynamics of our model provide a good estimate of the time course of the postsynaptic events that lead to synaptic plasticity in the striatum during procedural learning. To our knowledge, this is the first model that accounts for the effects of feedback delays on procedural learning.

The model outlined in this article is a computational cognitive neuroscience (CCN) model (Ashby and Helie, 2011). CCN models are similar to traditional cognitive models in the sense that a fundamental goal is to model behavior. However, CCN models account for behavior with an architecture that is constrained by known neuroanatomy and with dynamics that are constrained by known neurophysiology. Furthermore, learning in CCN models is constrained by the current literature on synaptic plasticity (e.g., LTP, LTD). In fact, a good CCN model makes no assumptions that are known to contradict the current neuroscience literature (i.e., the neuroscience ideal) and should provide a good fit of behavioral data and at least some neuroscience data. The model outlined in this article meets these criteria. The numerical values of all parameters in the category-learning model were set to the values used by Izhikevich (2007) and Ashby and Crossley (2011), and the neural architecture was constructed in accordance with a large body of neuroscience data. Thus, only a small number or additional parameters were estimated (e.g., Equation 1 learning parameters; the lag for generating the MSN alpha function, and the variance of the white noise in the premotor units), and all but the variance of motor noise was held fixed across all three experiments. Thus, the model is highly constrained yet provides an excellent account of the behavioral feedback delay data.

The current model is a model of the procedural learning system, which is the optimal system for learning II categories. According to the neurobiologically inspired COVIS model of category learning (Ashby et al., 1998), two systems operate during category learning. One is an explicit system that tests explicit hypotheses about category membership. The explicit system relies on working memory and executive attention and is mediated by the anterior cingulate, prefrontal cortex, the head of the caudate nucleus, and the hippocampus. The second system is the procedural-learning system modeled in this article. According to this account, both systems depend on the perceptual representation memory system, since both rely critically on input from visual cortical areas. COVIS assumes that these two systems compete on a trial-by-trial basis and that there is an initial bias toward the explicit system that can be overcome in cases when no explicit strategies yield adequate accuracy. A CCN model of the explicit system has not been fully implemented but much progress on many aspects of this model have been made (Ashby et al., 2005; Hélie and Ashby, 2009). Future work should complete a CCN model of the explicit system and combine that model with the current procedural-learning model.

This article proposes a neurobiologically detailed theory of procedural learning that is sensitive to varying feedback delays. The theory assumes that procedural learning is mediated by plasticity at cortical-striatal synapses that are modified by dopamine-mediated reinforcement learning. The model captures the time-course of the biochemical events in the striatum that cause synaptic plasticity, and thereby accounts for the empirical effects of various feedback delays on II category learning.

## Author notes

This research was supported in part by AFOSR grant FA9550-12-1-0355 to W. Todd Maddox and F. Gregory Ashby.

### Conflict of interest statement

The authors declare that the research was conducted in the absence of any commercial or financial relationships that could be construed as a potential conflict of interest.

## Footnotes

^1^The critical step in this DA induced cascade may be the phosphorylation of DARPP-32 (Dopamine and cAMP-Regulated Phosphoprotein) because the phosphorylated version of DARPP-32 deactivates proteins (e.g., PP-1) that reduce the LTP effects of CaMKII. Thus, when dopamine binds to D1 receptors an important LTP inhibiting action is reduced.

^2^The code of this procedural model is provided online: https://labs.psych.ucsb.edu/ashby/gregory/numrout.htm

^3^We chose the noise standard deviation to be 10% of the stimulus range on each dimension, since this value was typical of the parameter estimates found during decision bound modeling. Including this amount of noise reduces the accuracy of the best possible rule by about 2–4%. We did not explore any other noise variance values because increasing or decreasing the noise standard deviation even by 50% changes our overall predictions only negligibly.

^4^Unfortunately, the individual participant data from Maddox et al. (2003) are no longer available, so this approach is not possible.
