# Supplementary material for: A computational model of the temporal dynamics of plasticity in procedural learning: sensitivity to feedback timing
Source: Front Psychol. 2014 Jul 2;5:643. doi: 10.3389/fpsyg.2014.00643 (PMC4079082; doi:10.3389/fpsyg.2014.00643)
Supplement: Supplementary file 1 [file DataSheet1.DOCX]

**Appendix: Simulation Methods (Category-Learning Model)**

All model simulations were based on the network structure shown in Figure 2, which is a simplified version of the model described by Ashby and Crossley ([2011](#_ENREF_3)). The activation of all visual cortical units in this model is either off (with activation 0) or on (with activation 600) during the duration of stimulus presentation. The model of changes in the membrane potential of the MSNs, which was adapted from Izhikevich ([2007](#_ENREF_23)), includes two coupled differential equations for each MSN. The first models fast changes in membrane potential (measured in mV), and the second models slow changes in the activation and inactivation of various intracellular ion channels (e.g., Na^+^ and K^+^). We supplement the Izhikevich ([2007](#_ENREF_23)) model by assuming that the key inputs to the MSNs include excitatory inputs from sensory cortex and inhibitory input from other MSNs. Specifically, our complete MSN model assumes that the membrane potential in striatal unit A (for example) at time t, denoted S_A_(t), is determined by:

 (A.1)

$100\frac{du(t)}{dt}=-20\left[S_{A}(t)+80\right]-u(t)$ (A.2)

 (A.3)

where the constants *E* = 100 and σ_S_ = 20, I(t) is the input, and ε(*t*) is white noise. To produce spikes, when *S*_A_(*t*) = 40 mV then *S*_A_(*t*) is reset to *S*_A_(*t*) = -55 mV. Equation A.2 models the slow changes in various intracellular ion channels. When Equation A.1 produces a spike (i.e., when *S*_A_(*t*) = 40 mV), *u*(*t*) is reset to *u*(*t*) + 150. All specific numerical values in Equation A.1 and A.2 and the numerical values used in the resetting procedures are taken from Izhikevich ([2007](#_ENREF_23)).

The input, *I*(*t*) is defined in Equation A.3, where the constant γ_S_ = 100, *I_K,A_*(*t*) is the input (in this case, a square wave from visual cortex) and *w_K,A_*(*n*) is the strength of the synapse between sensory cortical unit K and striatal unit A on trial *n*, and its initial value is set to a random number drawn from a uniform distribution between 0.2 and 0.225 on trial 1. The second term on the right models lateral inhibition from striatal unit B. The function *f* [*S*_B_(*t*)] is called the alpha function and is a standard method for modeling the postsynaptic effects of a spike that occurs in a presynaptic neuron ([e.g., Rall, 1967](#_ENREF_30)). Every time the pre-synaptic neuron spikes, the following input is delivered to the postsynaptic neuron:

 (A.4)

This function has a maximum value of 1.0 and it decays to .01 at *t* = 7.64λ. The λ was set to 100. For example, *f* [*S*_B_(*t*)] models the postsynaptic effects of spikes produced in the presynaptic neuron, S_B_(t).

For all other units in the model, we exclude the slow regulatory term *u*(*t*), and instead model membrane potential with the standard quadratic integrate-and-fire model ([Ermentrout, 1996](#_ENREF_17)). For example, activation in the globus pallidus at time *t*, denoted by *G_J_*(*t*) (where *J* indicates either unit A or B), is described by:

(A.5)

where the constant α_G_ = 0.4175. The first term models the inhibitory input from the striatum, the second term ensures a high tonic firing rate, and the last term is the quadratic integrate-and-fire component that is the same as in Equation A.1. Spikes are produced after *G_J_*(*t*) = 35 by resetting to *G_J_*(*t*) = -50. Activation in the thalamus, *V_J_*(*t*), is modeled in the same way, except α_C_ = 0.275, and the constant out front is equal to 1.

Activation in premotor cortical unit A at time *t*, denoted by *C_A_*(*t*), is given by:

$100\frac{dC_{A}(t)}{dt}=\beta_{C}f\left[V_{A}(t)\right]-\gamma_{C}f\left[C_{B}(t)\right]+69+.7\left[C_{A}(t)+60\right]\left[C_{A}(t)+40\right]+\sigma_{C}\varepsilon(t)$ (A.6)

where the constants β_C_ = 2, γ_C_ = 1.25, and σ_C_ = 20 (for all except, for the 2003 experiment, σ_C_ = 380, because of the lower performance due to additional noise from masks), and ε(*t*) is white noise. The input *V*_A_(*t*) is from thalamus unit A. As in other units, spikes are produced when *C_J_*(*t*) = 35 by resetting to *C_J_*(*t*) = -50. The second term on the right models lateral inhibition in the same way as in Equation A.1. In tasks with two possible responses, evidence suggests that cortical units in premotor areas are sensitive to the cumulated difference in evidence favoring the two alternatives ([e.g., Shadlen & Newsome, 2001](#_ENREF_38)). We use a more biologically plausible method that is known to simulate this difference process – that is, we place a separate threshold on the activation of each unit, but include lateral inhibition between the units ([Usher & McClelland, 2001](#_ENREF_42)).

Finally, we model DA neuron firing using the Izhikevich ([2007](#_ENREF_23)) model of the regular spiking neuron:

$100\frac{dD(t)}{dt}=500I(t)+\left[D(t)+60\right]\left[D(t)+40\right]+E-u(t)+\sigma_{S}\varepsilon(t)$ (A.7)

$333\frac{du(t)}{dt}=-2\left[S_{A}(t)+60\right]-u(t)$ (A.8)

where the constants *E* = 71 and σ_S_ = 0.001, *I*(*t*) is the input, and ε(*t*) is white noise. To produce spikes, when *D*(*t*) = 35 mV then *D*(*t*) is reset to *D*(*t*) = -50 mV. When Equation A.7 produces a spike (i.e., when *D*(*t*) = 35 mV), *u*(*t*) is reset to *u*(*t*) + 100. All specific numerical values in Equations A.7 and A.8 and the numerical values used in the resetting procedures are taken from Izhikevich ([2007](#_ENREF_23)).

We produced the postsynaptic effects of MSN and DA units by generating an alpha function each time a spike was produced. In the case of DA, the mean basal firing rate is a positive constant. We subtracted this mean basal firing rate from the DA alpha function in order to have a magnitude around zero when input to DA neurons was zero. This way, when MSN and DA alpha functions were multiplied [*S_J_D*(*n*)], the product would also be zero during basal DA firing. The parameters in producing these alpha functions were decay, λ, (λ_MSN_ = 200, λ_DA_ = 100), and lag (i.e., time to generate the function after spiking started; lag_MSN_=550, lag_DA_=0);

**Table A1. Free Parameters for the Simulations.**

| ***Parameters*** | ***Experiment 1, 2, 3*** |
| --- | --- |
| *Equation 1: α_w_* | 1.5×10^-9^ |
| *Equation 1: β_w_* | 0.9×10^-9^ |
| *Equation 1: γ_w_* | 0.005×10^-9^ |
| *Equation 1: θ_NMDA_* | 4 |
| *Equation 1: θ_AMPA_* | 0.2 |
| *Alpha-function:* lag_MSN_ | 550 |
| *Equation A.6*: σ_C_ | 380, 20, 20 |
